# The Third Generation Anti-HER2 Chimeric Antigen Receptor Mouse T Cells Alone or Together With Anti-PD1 Antibody Inhibits the Growth of Mouse Breast Tumor Cells Expressing HER2 *in vitro* and in Immune Competent Mice

**DOI:** 10.3389/fonc.2020.01143

**Published:** 2020-07-14

**Authors:** Panyuan Li, Lingcong Yang, Tong Li, Shufang Bin, Bohao Sun, Yuting Huang, Kaiyan Yang, Daming Shan, Haihua Gu, Hongzhi Li

**Affiliations:** ^1^Zhejiang Provincial Key Laboratory of Medical Genetics, Key Laboratory of Laboratory Medicine, Ministry of Education, Wenzhou Medical University, School of Laboratory Medicine and Life Sciences, Wenzhou Medical University, Wenzhou, China; ^2^The Third People's Hospital of Dalian, Dalian, China; ^3^The First Affiliated Hospital of Wenzhou Medical University, Wenzhou, China

**Keywords:** HER2, breast cancer, chimeric antigen receptor (CAR)-T cells, PD1, immunotherapy

## Abstract

Chimeric Antigen Receptor (CAR)-T cells have great efficacy against CD19^+^ leukemia but little success for solid tumors. This study explored the effectiveness of third generation anti-HER2 CAR-T cells alone or in combination with anti-PD1 antibody on breast tumor cells expressing HER2 *in vitro* and in immune competent mouse model. The PDL1-positive mouse mammary tumor cell line 4T1 engineered to express luciferase and human HER2 was used as the target cell line (4T1-Luc-HER2). Anti-HER2 CAR-T cells were generated by transducing mouse spleen T cells with recombinant lentiviruses. ELISA analysis showed that IL-2 and IFN-γ secretion was increased in CAR-T cells co-cultured with the target cells, and the secretion of these two cytokines was increased further with the addition of anti-PD1 antibody. Lactate dehydrogenase assay revealed that CAR-T cells displayed a potent cytotoxicity against the target cells, and the addition of anti-PD1 antibody further enhanced the cytotoxicity. At the effector: target ratio of 16:1, cytotoxicity was 39.8% with CAR-T cells alone, and increased to 49.5% with the addition of anti-PD1 antibody. In immune competent syngeneic mouse model, CAR-T cells were found to be present in tumor stroma, inhibited tumor growth and increased tumor apoptosis significantly. Addition of anti-PD1 antibody further enhanced these anti-tumor activities. Twenty-one days after treatment, tumor weight was reduced by 50.0% and 73.3% in CAR-T group and CAR-T plus anti-PD1 group compared with blank T group. Our results indicate that anti-PD1 antibody can greatly increase the efficacy of anti-HER2 CAR-T against HER2-positive solid tumors.

## Introduction

Breast cancer has the highest incidence in women worldwide (1). About 20–25% of breast cancers are human epidermal growth factor receptor-2 (HER2) positive due to HER2 amplification or HER2 overexpression (2). Patients with HER2-positive breast cancer were not sensitive to chemotherapy and endocrine therapy, having a poor prognosis (3). Other malignant solid tumors with HER2 overexpression include ovarian cancer, non small cell lung cancer, prostate cancer, gastric cancer, colorectal cancer, renal cell carcinoma, and bladder cancer (4–6). HER2 is a significant target for cancer therapy (7–10). Herceptin (trastuzumab) is a humanized monoclonal antibody targeting HER2 and is widely used for the treatment of HER2-positive breast cancer. However, the efficacy of herceptin is limited as the resistance to its action frequently develops (11).

Treating solid tumors with chimeric antigen receptor (CAR)-T cells is being actively explored (12–16). In this immunotherapeutic strategy, T cells isolated from patients are genetically modified to express CAR on the cell surface. CAR contains the extracellular domain from the single-chain variable fragment (scFv) region of the antibody for tumor associated antigen, the transmembrane domain, and the intracellular domain from the signal activating regions for T cells. CAR-T cells recognize target antigen on the surface of tumor cells without the help of MHC molecules. The first generation CAR consists of an antigen recognition scFv and the CD3ζ signaling region whereas the second generation CAR includes an additional signaling region from one co-stimulatory molecule and the third generation CAR includes additional signaling regions from two co-stimulatory molecules (17).

Although CAR-T cell therapy has shown great success against hematological malignancies, it is less effective in the treatment of solid tumors (18–20). There are many factors limiting the anti-tumor effect of CAR-T cells (20–22). They includes the increased expression of inhibitory immune receptors such as programmed death-1 (PD1/CD279), which limits the strength and duration of CAR-T cell activation (23). PD1 expression is increased following the binding of T-cell receptor with its ligand. The ligand for PD1 is programmed cell death 1 ligand 1 (PDL1/CD274) that is also overexpressed in various cancers (24). The PD1/PDL1 pathway was regarded as a promising target for cancer therapy (25, 26). In recent years, it has been reported that CAR-T cell therapy and PD1 checkpoint blockade is one of the reasonable combinations in immunotherapy of solid tumor models (27–30).

We have previously designed and constructed a third-generation anti-HER2 CAR that carried two co-stimulatory molecules (CD28 and CD137). The third generation anti-HER2 CAR or anti-HER2 CAR-T cells in combination with anti-PD1 antibody have not been tested in clinical trials for breast cancer. We hypothesized that anti-HER2 CAR-T cells alone should have efficacy against HER2^+^ breast cancer cells. Anti-HER2 CAR-T cells in combination with anti-PD1 antibody could overcome immunosuppression of anti-HER2 CAR-T cells induced by PD1/PDL1 pathway, and enhance the therapeutic efficacy. In this study, we generated the mouse breast cancer cell line 4T1-Luc-HER2 and used it as HER2 and PDL1 double positive target cells. We made anti-HER2 CAR-T cells by transducing mouse T cells with CAR-recombinant lentivirus. Using our tumor target cells and anti-HER2 CAR-T cells, we evaluated the anti-tumor effect of CAR-T cells *in vitro*, with or without anti-PD1 antibody. In addition, we established a new HER2 and PDL1 double positive breast cancer mouse model for the novel purpose of testing anti-tumor effect of CAR-T cells plus anti-PD1 in a complete tumor-immune system microenvironment *in vivo*.

## Materials and Methods

### Cell Lines and Media

Mouse breast cancer cell line 4T1 cells were grown in RPMI-1640 medium (Gibco, California, USA) supplemented with 10% fetal bovine serum (FBS) (Gibco). Human breast cancer cell line BT474 and SKBR3 cells, and human embryonic kidney cell line HEK 293T-17 cells were grown in Dulbecco's modified Eagle's medium (DMEM) (Gibco) supplemented with 10% FBS. All the above cell lines were bought from ATCC (American Type Culture Collection, Manassas, VA, USA) and cultured with 5% CO_2_.

### Generation of the Mouse Breast Cancer 4T1-Luc-HER2 Cells Expressing Both Luciferase (Luc) and HER2

Since there is no HER2-positive/luciferase-positive mouse breast cancer cell line commercially available, we generated 4T1-Luc-HER2 cells from HER2-negative/luciferase-negative mouse breast cancer cell line 4T1 cells as stated below. The retroviral expression plasmid pLNCX2-GFP-Luc was kindly provided by Dr. Steve Anderson (University of Colorado, School of Medicine), in which GFP and luciferase was fused (31). For generating GFP-Luc-recombinant retrovirus, 293T-17 cells were co-transfected using polyethyleneimine (PEI) with four plasmids, including pLNCX2-CMV-GFP-Luc, pVSV-G, pJK3 and pTAT2 (SBI System Biosciences, Palo Alto, CA, USA) in the ratio of 2:1:1:1. The lentiviral expression plasmid pLEX-CMV-HER2 was kindly provided by Dr. Bolin Liu (University of Colorado, School of Medicine) (32). For generating humanized-HER2-recombinant lentivirus, 293T-17 cells were co-transfected using PEI with three plasmids, including pLEX-CMV-HER2, pSPAX2 and pMD2G (SBI System Biosciences, Palo Alto, CA, USA) in the ratio of 4:3:1. The retrovirus or lentivirus supernatants were collected at 48 and 72 h post-transfection, centrifuged at 3,000 rpm for 10 min and filtered through a 0.45 μm membrane before stored at −80°C.

The luciferase-negative/HER2-negative mouse breast cancer 4T1 cells were cultured with GFP-Luc-recombinant retrovirus supernatants supplemented with 8 μg/ml of polybrene (Sigma, St Louis, MO, USA) for 16 h. Twenty-four hours later, 4T1 expressing luciferase was selected with 600 μg/ml of G418 (Solarbio, Beijing, China) for 1 week. Then, the selected cells were incubated with HER2-recombinant lentivirus supernatants supplemented with 8 μg/ml of polybrene, and selected with 2.5 μg/ml puromycin (Invitrogen, San Diego, USA) for 4 days. Finally, the stable 4T1-Luc-HER2 cells expressing both luciferase and HER2 were generated.

### Generation of the Third-Generation Anti-HER2 Mouse CAR-T Cells

The third generation anti-HER2 CAR was generated according to our published work (33). The recombinant lentivirus expressing the anti-HER2 CAR was produced by co-transfecting 293T-17 cells with recombinant lentiviral vector pLVX-EF1α-CAR-IRES-ZsGreen1 together with the packaging plasmids pSPAX2 and pMD2.G. The protocol for packaging, concentration and purification of the recombinant lentivirus was essentially as we described previously (33).

Splenocytes were from 6-week female BALB/c mice. Mouse spleen lymphocytes were isolated by density-gradient centrifugation (at 900 × g) using a lymphocyte separation medium Histopaque (Sigma). Mouse spleen lymphocytes were activated in 24-well plates at a density of 1 × 10^6^/ml by 5 μg/mL of plate-bound anti-CD3 monoclonal antibody (Invitrogen) and 2 μg/mL of solution-state anti-CD28 monoclonal antibody (Invitrogen) for 3 days. Mouse T cells were cultured in RPMI 1640 medium with 10% FBS, 0.2 mmol/L L-glutamine (Gibco), 50 μmol/L β-mercaptoethanol (Gibco), 25 mmol/L HEPES (Gibco), and 500 IU/mL recombinant human interleukin 2 (IL-2) (PEPROTECH, Rocky Hill, NJ, USA).

On day 4, the CD3^+^ mouse splenic T cells were mixed with CAR-recombinant lentiviruses at a multiplicity of infection (MOI) value of 20 in the presence of 8 μg/mL polybrene. The tissue culture plate was then centrifuged at 1,500 × g for 2 h at 32°C, incubated for 10–14 h at 37°C, replaced with fresh medium and cultured for an additional 3–4 days for expansion. Transduced cells were examined for proper GFP expression rate (~40% GFP-positive cell ratio) under fluorescence microscope (Nikon Eclipse Ti), and subjected to flow cytometry to evaluate the GFP (CAR) positive cell percentage and western blot for CAR expression level, respectively.

### Flow Cytometric Analysis

The expression of HER2 on 4T1-Luc-HER2 cells was detected using allophycocyanin (APC) labeled anti-human CD340 (erbB2/HER2) antibody, with APC mouse IgG1κ (both from BioLegend, San Diego, CA, USA) used as an isotype control. The expression of PDL1 on 4T1-Luc-HER2 cells was determined using PE/Cy7 anti-mouse CD274 (PDL1) antibody (BioLegend), with PE/Cy7 Rat IgG2bκ (BioLegend) used as an isotype control. The expression of CD3 in the activated mouse T cells was evaluated using APC anti-mouse CD3ε (BioLegend), with APC Armenian Hamster IgG (BioLegend) used as an isotype control. 7-aminoactinomycin D (7-AAD) solution (BioLegend) was used to determine the percentages of viable CAR-T cells. The expression of PD1 on anti-HER2 CAR-T cells was examined using PE-conjugated anti-mouse PD1 antibody (BD Biosciences), with PE mouse IgG2aκ (BD Biosciences) as an isotype control.

### Western Blot Analysis

Western blot was used to detect the expression level of HER2 in 4T1-Luc-HER2 cells and the expression level of CAR in the CAR-transduced T cells. Cells were lysed in lysis buffer, and the protein concentrations of cell lysates were analyzed by the BCA kit (Beyotime Institute of Biotechnology, Shanghai, China). Lysates (10 μg per lane) were resolved by 10% SDS-PAGE, transferred to PVDF membranes (EMD Millipore). The blots were blocked with 5% non-fat dry milk in TBST, immunoblotted with anti-human HER2 antibody (dilution 1:1,000; Abcam) or anti CD3ζ polyclonal antibody (dilution 1:1,000; Affinity Biosciences, Jiangsu, China) and anti-rabbit HRP-conjugated secondary antibody (dilution 1:5,000; Santa Cruz Biotechnology, Inc. CA, USA), developed utilizing ECL reagent (Beyotime Institute of Biotechnology), and detected using the ChemiDoc MP Imaging System (Bio-Rad Laboratories, Inc.). The expression level of HER2 or CAR (containing exogenous CD3ζ) was detected, respectively, using tubulin or endogenous CD3ζ as a loading control.

### Detect the Expansion of CAR-T Cells

The blank T cells or anti-HER2 CAR-T cells were co-cultured with HER2^−^ 4T1 or HER2^+^ 4T1-Luc-HER2 tumor cells at an effector: target ratio of 4:1 in the absence or presence of 20 μg/mL anti-PD1 antibody. The T cells were counted using hemocytometer with trypan blue exclusion, respectively, at 24, 48, and 7 2h after co-cultured with tumor cells.

### Detection of Cytokine Secretion by the CAR-T Cells

Anti-HER2 CAR-T cells or in the presence of 20 μg/ml anti-PD1 antibody were incubated with HER2^+^ 4T1-Luc-HER2 cells at an effector: target ratio of 4:1 for 24 h in a 96-well plate. Anti-PD1 antibody (clone RMP1-14) was purchased from BioXCell (New Hampshire, USA). ELISA kits (R&D Systems, Inc. Minneapolis, USA) were used to analyze mouse IL-2 and IFN-γ concentrations in the supernatants of T cells according to the manufacturer's instructions. The co-cultures of HER2^−^ 4T1 cells with anti-HER2 CAR-T cells (or in combination with anti-PD1 antibody), or HER2^+^ 4T1-Luc-HER2 cells with the blank T cells (or in combination with anti-PD1 antibody) were used as negative controls.

### *In vitro* Cytotoxicity Assay

The cytotoxicity assay was essentially as described (34). Briefly, anti-HER2 CAR-T cells (effector cells) in the absence or presence of 20 μg/mL anti-PD1 antibody were incubated with HER2^+^ 4T1-Luc-HER2 cells (target cells) at the effector: target ratios of 2:1, 4:1, 8:1, and 16:1 for 18 h in a 96-well plate. The co-cultures of anti-HER2 CAR-T cells in the absence or presence of anti-PD1 antibody with HER2^−^ 4T1 cells, the blank T cells in the absence or presence of anti-PD1 antibody with HER2^+^ 4T1-Luc-HER2 cells were used as negative controls. Specific lactate dehydrogenase (LDH) released into the cell-free supernatant from the target cells was determined using the cytotoxicity LDH detection kit (Genmed, Addlestone, UK) according to the manufacturer's instructions. The amount of released LDH was used to assess the extent of target cell lysis, which can be translated into the effectiveness of effector cells. Percent cytotoxicity was calculated according to OD values utilizing the following formula: Cytotoxicity (%) = (Experimental lysis − Effector spontaneous lysis − Target spontaneous lysis)/(Target maximum lysis − Target spontaneous lysis) ×100%.

### Construction of the Syngeneic Mammary Tumor Model

Protocols for the animal studies were approved by the Institutional Animal Care and Use Committee of Wenzhou Medical University. All animal experiments were performed in accordance with the relevant guidelines and regulations. BALB/c female mice with an intact immune system (6-week-old, weighed 17–20 g) purchased from GemPharmatech (Nanjing, China) were used for *in vivo* experiments. After 1 week of housing in the animal facility, 4 × 10^6^ of 4T1-Luc-HER2 cells in 0.1 mL PBS mixed with 0.1 mL matrigel (Corning, Bedford, MA, USA) were injected subcutaneously into each of 24 mice at right back region on day 0. On day 14, when the diameter of the engrafted tumors reached about 6 mm, mice were randomized into four groups for treatments. The experiment was repeated for three times.

### Anti-tumor Treatments *in vivo*

Twenty-four tumor-grafted mice were randomly divided into four groups (*n* = 6): blank T group (injection of blank T cells), anti-PD1 group (injection of anti-PD1 antibody), CAR-T group (injection of CAR-T cells), CAR-T plus anti-PD1 group (injection of CAR-T cells and anti-PD1 antibody). The tumor-grafted mice were administrated via caudal vein injection with blank T cells or CAR-T cells, 1 × 10^7^ cells in 0.1 mL PBS/each mouse/each time, on day 14 and 21. The tumor-grafted mice were administrated intraperitoneally with anti-PD1 antibody, 250 μg in 0.1 mL PBS/each mouse/each time on day 14, 18, 22, and 26. All the mice were injected intraperitoneally with 20,000 IU IL-2 once every 2 days from day 14 to day 34.

### Monitoring Tumor Growth and Collecting Tumor Tissues

Tumor growth was monitored on day 14 (just before anti-tumor treatment) and 28 (after anti-tumor treatment for 14 days) using Lumina Series III IVIS imaging system (PerkinElmer, MA, USA) as described (35). Briefly, on the day of IVIS imaging, mice were first anesthetized with isoflurane (RWD Life Science, Shenzhen, China) and then injected with 150 mg/kg luciferase solution (PerkinElmer) intraperitoneally. Images were captured using the IVIS system and analyzed with the Living Image @4.3.1. software.

During the above anti-tumor experiment *in vivo*, tumor volumes were measured once every 5 days using a caliper, and calculated using the equation V (mm^3^) = 1/2 × L × W^2^, where L (length) is the largest diameter and W (width) is the smallest diameter.

On day 35 (after anti-tumor treatment for 21 days), all of the mice were euthanized. All tumors were excised from tumor-bearing mice, weighed, fixed, and embedded for pathologic examination and immunohistochemical analysis. The inhibition ratio of tumor weight (%) = (tumor weight of group 1 − tumor weight of group 2)/tumor weight of group 1 × 100%.

### Histological Examination and Immunohistochemical Analysis of Tumor Tissues

Tumor sections (three for each tumor) were stained with hematoxylin-eosin (HE) as described (36). HE stained tumor tissues were examined with a microscope at 400 × magnification.

Tumor sections (five for each tumor) were immunostained with antibodies for cleaved caspase-3 and CD3 expression. Rabbit anti-mouse cleaved caspase-3 antibody and rabbit anti-mouse CD3ε antibody (1:200 dilution) were purchased from Cell Signaling Technology (Danvers, MA, USA). The sections were treated with antigen retrieval solution followed by 3% hydrogen peroxide treatment, blocked with 5% goat serum, incubated with primary antibody and with biotin-labeled secondary antibody (goat anti-rabbit; 1:1,000 dilution; Abcam, Cambridge, UK). The stained tissues were visualized by using the DAB chromogen (Abcam) reagent and counterstained with hematoxylin. For quantification of immunohistochemical staining, positive cell percentages were counted in five random 400 × microscopic fields for each tissue section.

### Statistical Analysis

Probability (*P*) values were calculated by using SPSS 22.0 software. Unpaired student T-test was used when comparing data between two groups. Comparisons among three or more groups were performed by one-way ANOVA. First homogeneity of variance was tested, if equal variances assumed, then *P*-values were calculated by Tukey; otherwise, *P*-values were calculated by Tamhane's T2. The difference with *P* < 0.05 was considered statistically significant.

## Results

### Successful Generation of the Mouse Breast Cancer 4T1-Luc-HER2 Cells Expressing Both Luciferase and HER2

**Figure 1:**
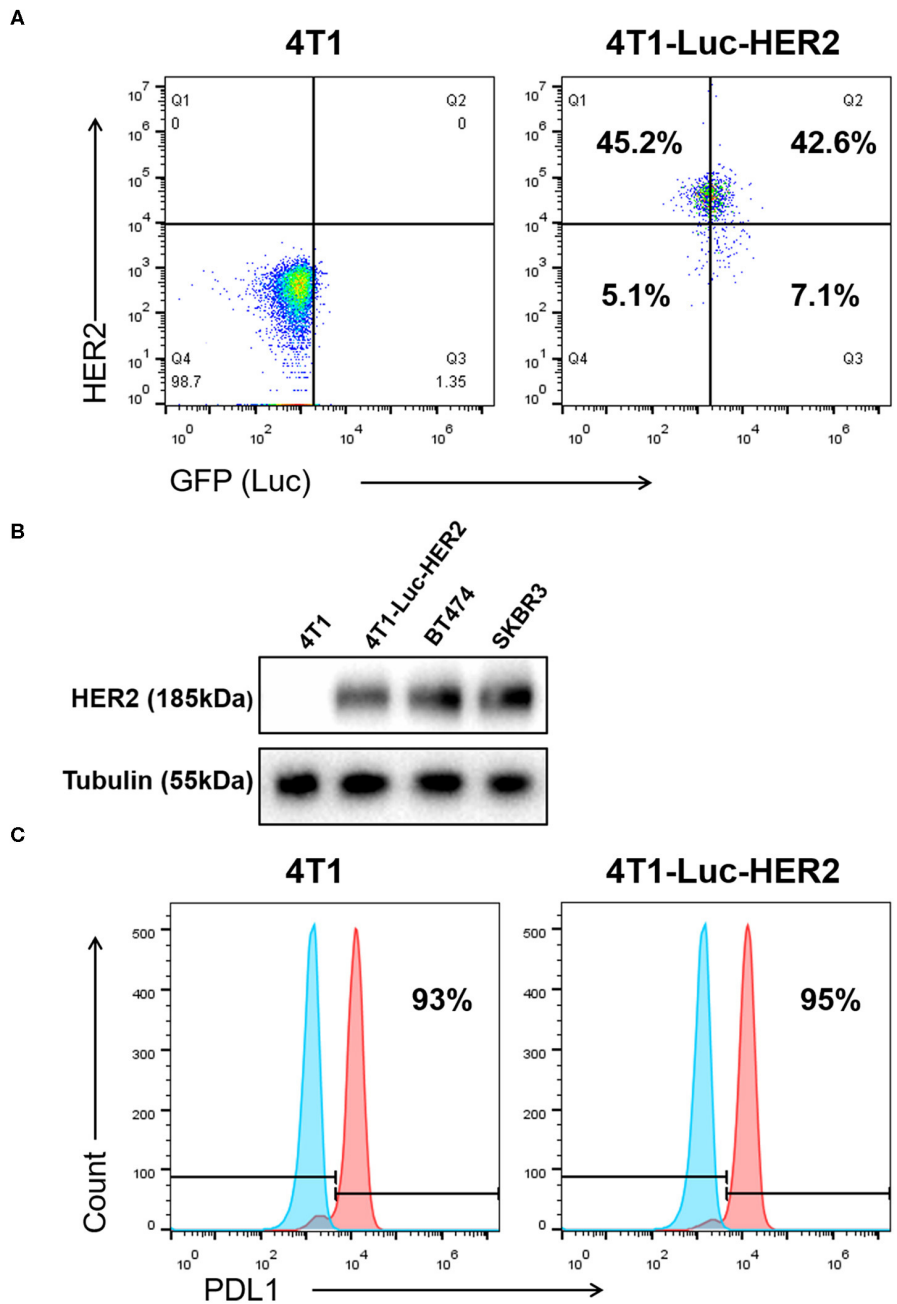
Expression of HER2, GFP (Luc) and PDL1 in 4T1-Luc-HER2 cells. **(A)** The expression level of HER2 and GFP (Luc) in 4T1-Luc-HER2 cells was detected by flow cytometry (using anti-human APC-HER2). 4T1 cells were set as HER2^−^/GFP (Luc)^−^ control. The values indicated the percentages of positive or negative cells. **(B)** The expression level of HER2 in 4T1-Luc-HER2 cells was detected by western blotting, using tubulin as a loading control. 4T1 cells were set as HER2-negative control. BT474 and SKBR3 cells were set as HER2-positive controls. **(C)** The expression level of PDL1 in 4T1-Luc-HER2 and 4T1 cells was detected by flow cytometry (using anti-mouse PE/Cy7-PDL1). The values indicated the percentages of PDL1-positive cells. The left histogram was an isotype control. The experiments have been repeated for three times. Luc, luciferase.

After the mouse breast cancer 4T1 cells were transduced with GFP-Luc-recombinant retroviruses and HER2-recombinant lentiviruses, the expression of both GFP (luciferase) and HER2 in 4T1-Luc-HER2 cells was detected by flow cytometry and western blot, using 4T1 cells as a negative control. The percentage of GFP (Luc)^+^ or HER2^+^ cells was 49.7 and 87.9%, respectively; the percentage of GFP (Luc)^+^/HER2^+^ double positive cells was 42.6%, detected by flow cytometry (Figure 1A). The expression level of HER2 in 4T1-Luc-HER2 cells was, at least, as same as that in two HER2-positive controls (human breast cancer BT474 and SKBR3 cells), detected by western blot (Figure 1B).

Before testing the efficacy of anti-HER2 CAR-T cells together with anti-PD1 antibody against breast cancer cells, we checked the expression of PDL1 on both 4T1 and 4T1-Luc-HER2 cells. The percentages of PDL1-positive 4T1 and 4T1-Luc-HER2 cells were 93 and 95%, respectively, detected by flow cytometry (Figure 1C).

### Generation of the Third Generation Anti-HER2 CAR-T Cells

**Figure 2:**
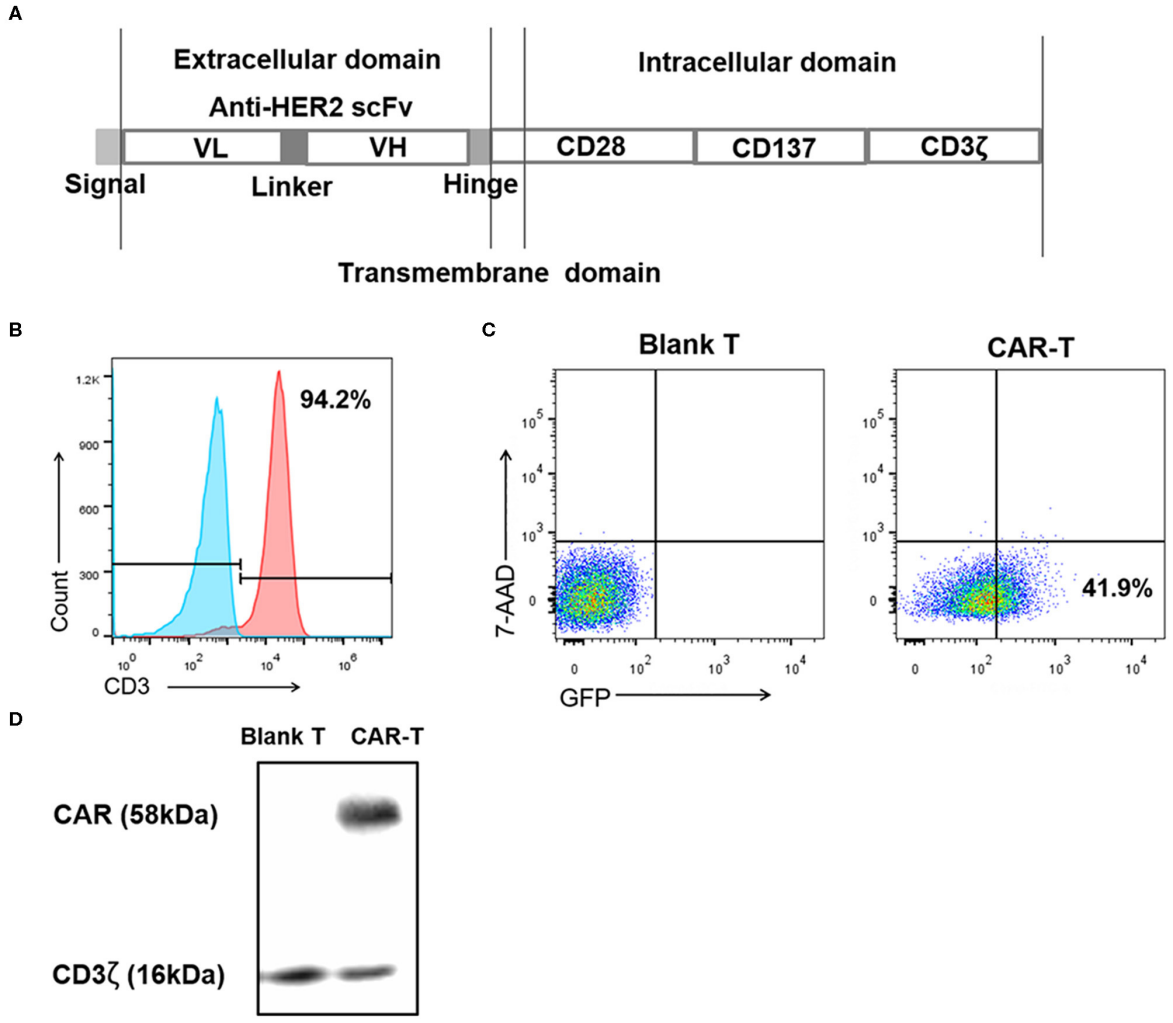
Preparation of the third generation anti-HER2 CAR-T cells. **(A)** Diagram of anti-HER2 CAR. **(B)** After activation and separation, mouse spleen lymphocytes were detected by anti-CD3 monoclonal antibody using flow cytometry. The values indicated the percentage of CD3-positive mouse spleen T cells. The left histogram was an isotype control. **(C)** After the mouse spleen T cells were transduced with the recombinant lentiviruses at MOI 20 for 4 days, the percentages of 7-AAD-negative (viable) cells and GFP-positive (transduced) cells were analyzed using flow cytometry, with (untransduced) blank T cells as the control. The value indicated the percentage of transduced viable cells. **(D)** The expression level of CAR (containing exogenous CD3ζ) in CAR-T cells was detected by western blotting using an anti-CD3ζ antibody, with blank T cells as negative control, and endogenous CD3ζ as a loading control. The experiments have been repeated for three times. CAR, chimeric antigen receptor; scFv, single chain variable fragment; VH, heavy chain variable region; VL, light chain variable region; MOI, multiplicity of infection; 7-AAD, 7-aminoactinomycin D; Blank T, T cells not transduced; CAR-T, T cells transduced with CAR.

The preparation of third generation anti-HER2 CAR-T cells started from the construction of anti-HER2 CAR as shown in Figure 2A, which was packed in lentivirus described in Materials and Methods above. The second step was preparation of mouse spleen T cells through activation of spleen lymphocytes and separation of spleen T cells. As detected by flow cytometry in Figure 2B, 94.2% of isolated cells were CD3-positive T cells. These cells were then transduced with CAR-recombinant lentivirus at a MOI of 20 for 4 days. GFP-positive (transduced) and 7-AAD-negative (viable) cells were counted by flow cytometer to evaluate the transduction efficiency. The transduction efficiency was 41.9% shown in Figure 2C. The expression level of CAR (containing exogenous CD3ζ) in transduced T cells was detected by an anti-CD3ζ antibody in western blotting. The endogenous CD3ζ was used as a loading control. CAR-T cells expressed the expected exogenous CD3ζ as part of CAR (58 kDa), and its expression level was much more than endogenous CD3ζ (16 kDa) (Figure 2D).

### PD1 Expression Is Increased in Anti-HER2 CAR-T Cells When Co-cultured With HER2-Positive Breast Cancer Cells

**Figure 3:**
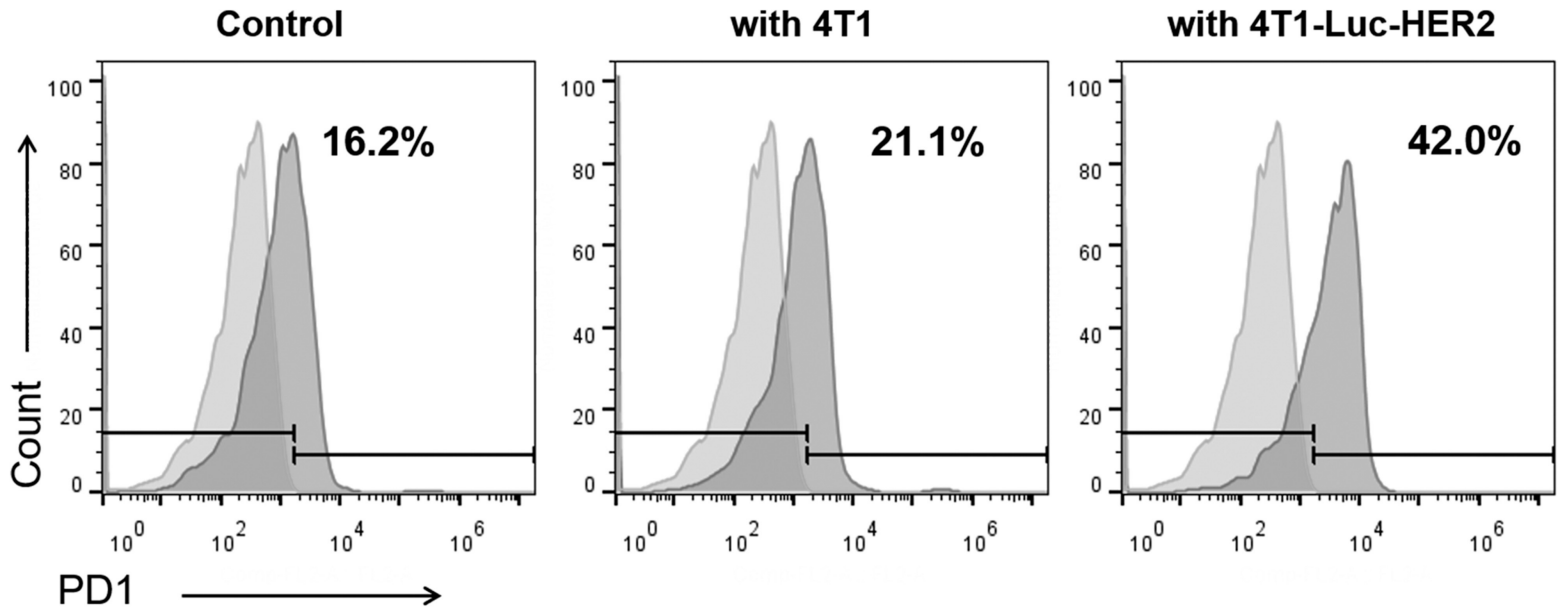
PD1 expression is increased in anti-HER2 CAR-T cells when co-cultured with HER2-positive breast cancer cells. Anti-HER2 CAR-T cells were cultured alone as control, or co-cultured with HER2^−^ 4T1 cells, or HER2^+^ 4T1-Luc-HER2 cells at an effector: target ratio of 4:1 for 24 h. Then, anti-HER2 CAR-T cells were subjected to flow cytometry analysis using PE anti-mouse PD1 antibody (right histogram) and PE isotype control (left histogram). The experiments have been repeated for three times.

It is known that after activation of T cells, the expression of inhibitory receptors including PD1 is increased. Therefore, we examined the PD1 expression in anti-HER2 CAR-T cells activated by HER2-positive breast cancer cells. Flow cytometry results demonstrated that 21.1% of the anti-HER2 CAR-T cells were PD1^+^ when co-cultured with HER2^−^ 4T1 cells (Figure 3). In contrast, PD1^+^ cells in anti-HER2 CAR-T cells were significantly increased to 42.0% when co-cultured with HER2^+^ 4T1-Luc-HER2 cells (Figure 3).

### HER2-Positive Breast Cancer Cells Induced Expansion of Anti-HER2 CAR-T Cells Can Be Further Enhanced With the Addition of Anti-PD1 Antibody

**Figure 4:**
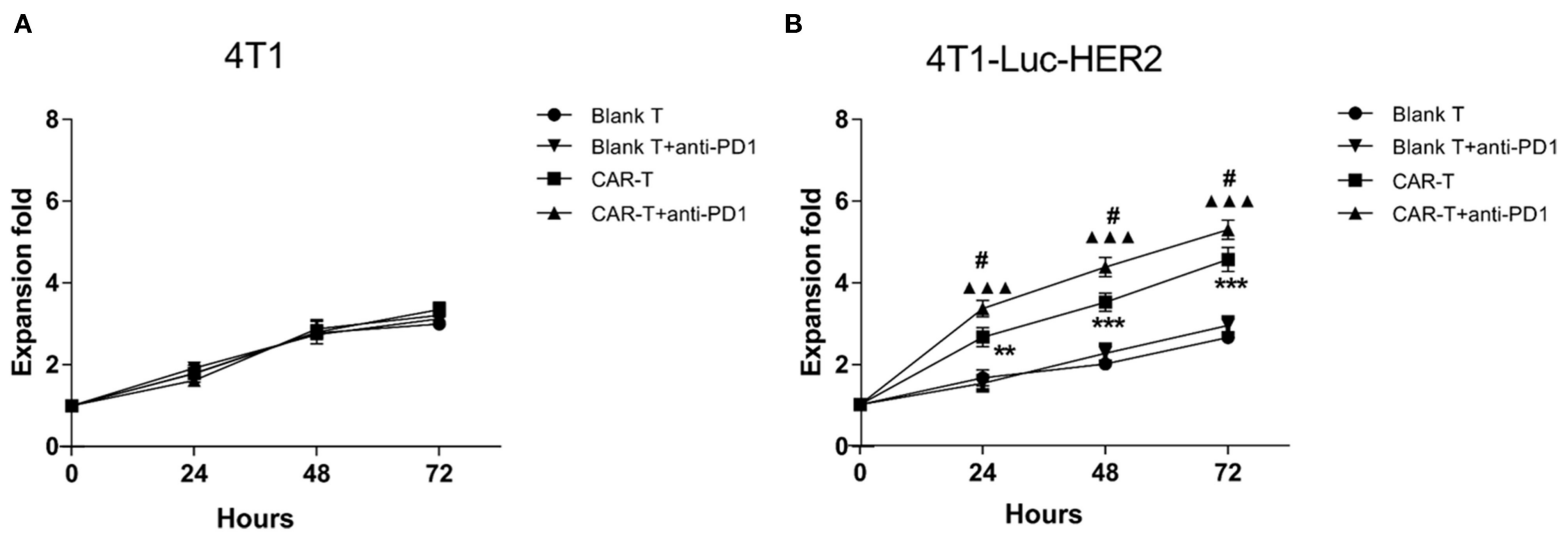
HER2-positive breast cancer cells induced expansion of anti-HER2 CAR-T cells can be further enhanced with the addition of anti-PD1 antibody. Blank T cells or anti-HER2 CAR-T cells were co-cultured with **(A)** 4T1 or **(B)** 4T1-Luc-HER2 cells in the absence or presence of anti-PD1 antibody at an effector: target ratio of 4:1 for 24, 48 or 72 h. Viable cells were counted by trypan blue exclusion each day. The mean values from three different experiments were presented. CAR-T group was compared with blank T group, **P < 0.01 and ***P < 0.001; or CAR-T plus anti-PD1 group was compared with blank T plus anti-PD1 group, ▴▴▴*P* < 0.001; or CAR-T group plus anti-PD1 group was compared with CAR-T group, ^#^*P* < 0.05.

T cells can proliferate violently after being activated by antigens presented by MHC molecules. We examined the proliferation of blank T or anti-HER2 CAR-T cells after they were co-cultured with HER2^−^/PDL1^+^ 4T1 or HER2^+^/PDL1^+^ 4T1-Luc-HER2 breast cancer cells at effector: target ratio of 4:1 in the absence and presence of anti-PD1 antibody for 24, 48, and 72 h. As a negative control, blank T cells or anti-HER2 CAR-T cells proliferated slowly when co-cultured with 4T1 cells regardless of the presence anti-PD1 antibody (Figure 4A). In contrast, the growth of anti-HER2 CAR-T cells was significantly higher than blank T cells when co-cultured with 4T1-Luc-HER2 cells (^**^P < 0.01 and ^***^P < 0.001) (Figure 4B). Importantly, the addition of anti-PD1 antibody further enhanced the growth of anti-HER2 CAR-T cells when co-cultured with 4T1-Luc-HER2 cells (^#^*P* < 0.05) (Figure 4B). It is suggested that the combination of CAR-T and PD1 blockade has an additive effect.

### Anti-HER2 CAR-T Cell Activation Induced by HER2-Positive Breast Cancer Cells

**Figure 5:**
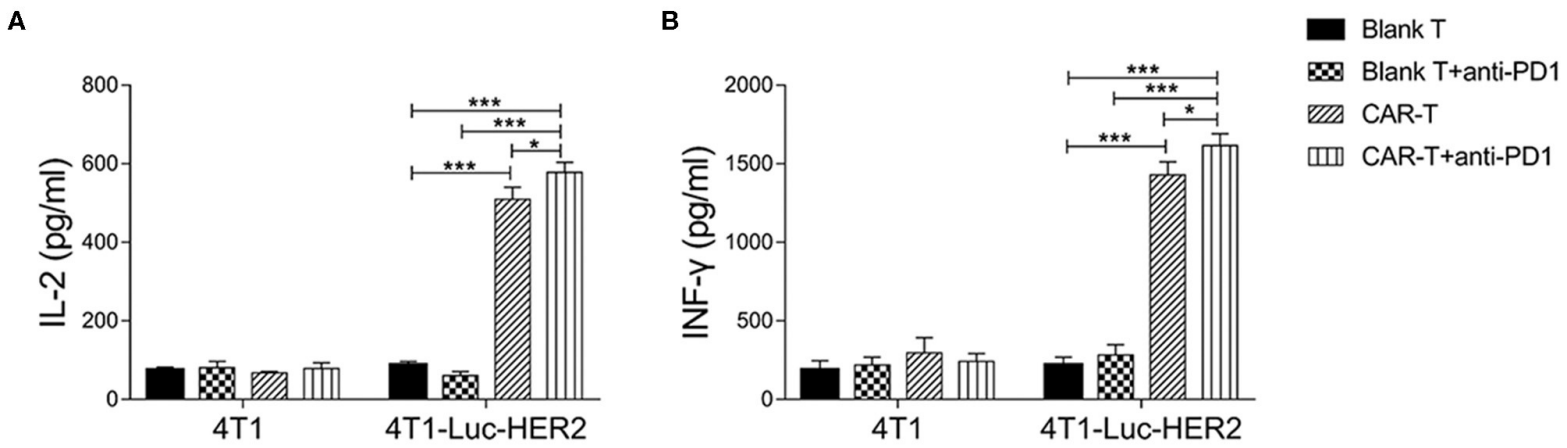
Cytokine secretion of anti-HER2 CAR-T cells. After anti-HER2 CAR-T cells (effector cells) (or in combination with anti-PD1 antibody) were co-cultured with HER2^+^ 4T1-Luc-HER2 cells (target cells) at an effector to target ratio of 4:1 for 24 h, supernatants were collected. ELISA kits for detecting **(A)** IL-2 and **(B)** IFN-γ were used to analyze supernatants. Supernatants from the co-culture of anti-HER2 CAR-T cells with HER2^−^4T1 cells, as well as from the co-culture of (untransduced) blank T cells (or in combination with anti-PD1 antibody) with HER2^+^ 4T1-Luc-HER2 cells were used as negative controls. Three repeated tests were performed and the mean values were presented. For **(A)** IL-2 or **(B)** IFN-γ secretion, CAR-T group was compared with blank T group; or CAR-T plus anti-PD1 group was compared with blank T plus anti-PD1 group; or CAR-T plus anti-PD1 group was compared with CAR-T group; or CAR-T plus anti-PD1 group was compared with blank T group (*P < 0.05; ***P < 0.001). CAR, chimeric antigen receptor; Luc, luciferase; CAR-T, T cells transduced with CAR; Blank T, T cells not transduced.

To evaluate whether anti-HER2 CAR-T cells were activated by target cells in the absence or presence of anti-PD1 antibody, cytokine secretion of IL-2 and IFN-γ from anti-HER2 CAR-T cells was detected by ELISA. The results revealed that IL-2 and IFN-γ secretion were significantly increased in CAR-T group when compared with blank T group (*P* < 0.001) after co-cultured with 4T1-Luc-HER2 cells at an effector to target ratio of 4:1 for 24h (Figure 5). IL-2 increased to 5.8-fold, and IFN-γ increased to 6.6-fold. IL-2 and IFN-γ secretion of CAR-T group co-cultured with 4T1-Luc-HER2 cells, increased to 7.6- and 4.8-fold respectively, compared with CAR-T group co-cultured with 4T1 cells. These results indicated that CAR-T cells could specifically bind to 4T1-Luc-HER2 cells and be activated. This activation could be enhanced further by adding anti-PD1 antibody as indicated by more IL-2 and IFN-γ secretion of columns representing CAR-T cells plus anti-PD1 antibody (*P* < 0.05) (Figure 5).

### Anti-PD1 Antibody Enhances the Cytotoxicity of Anti-HER2 CAR-T Cells Against HER2-Positive Breast Cancer Cells

**Figure 6:**
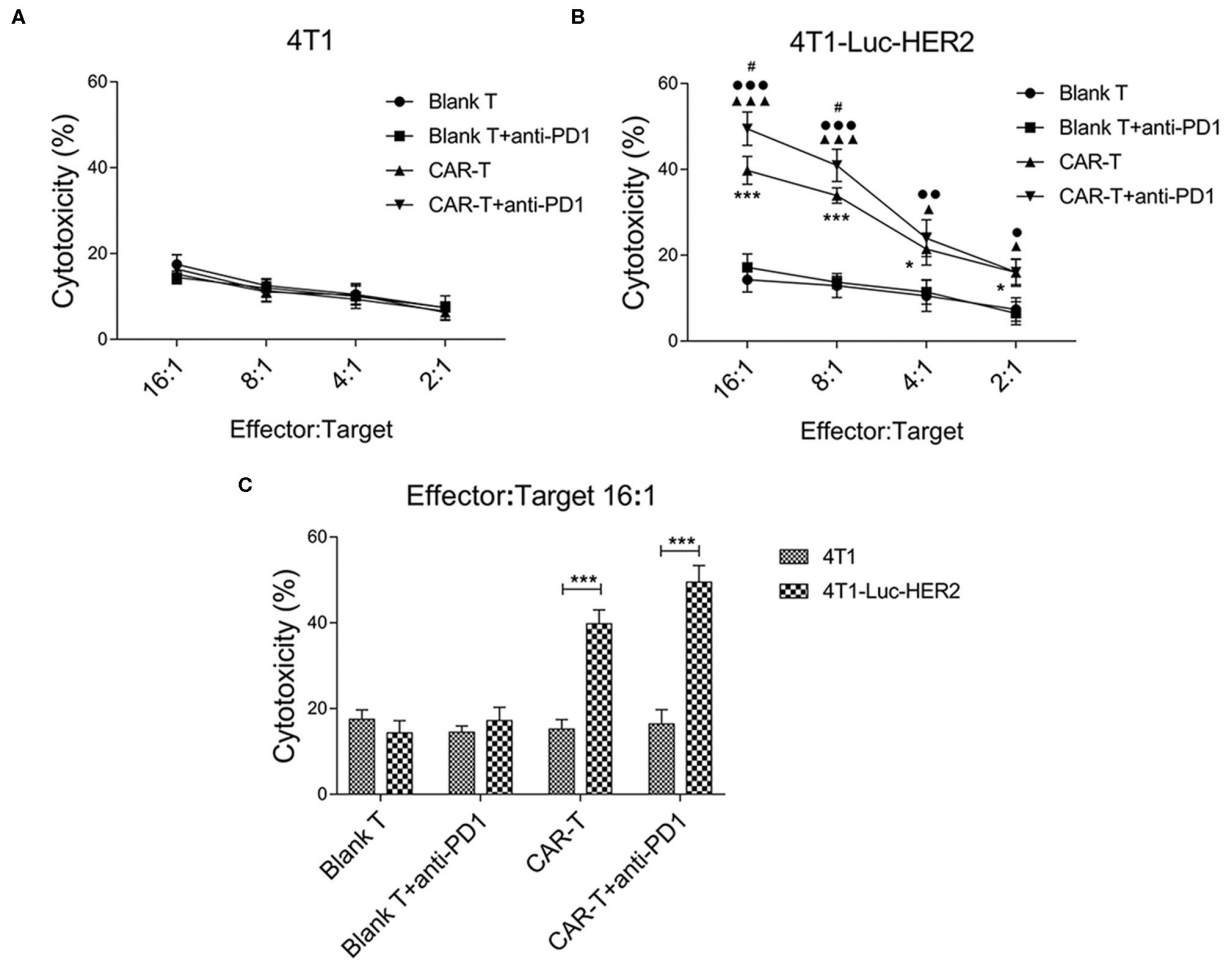
The cytotoxicity of anti-HER2 CAR-T cells (or in combination with anti-PD1 antibody) against 4T1-Luc-HER2 cells. **(A)** As a negative control, anti-HER2 CAR-T cells were co-cultured with HER2^−^ 4T1. **(B)** After anti-HER2 CAR-T cells (effector cells) were co-cultured with HER2^+^ 4T1-Luc-HER2 (target cells) (or in combination with anti-PD1 antibody) at different ratios of effector: target for 18 h, supernatants were detected using a cytotoxicity LDH detection kit for LDH released from lysed target cells. Cytotoxicity (%) was calculated. At effector: target ratios of 2:1, 4:1, 8:1 or 16:1 respectively, the cytotoxicity (%) of CAR-T group was compared with blank T group, *P < 0.05, ***P < 0.001; or CAR-T plus anti-PD1 group was compared with blank T plus anti-PD1 group, ▴*P* < 0.05, ▴▴▴*P* < 0.001; or CAR-T plus anti-PD1 group was compared with CAR-T group, #*P* < 0.05; or CAR-T plus anti-PD1 group was compared with blank T group, •*P* < 0.05, ••*P* < 0.01, •••*P* < 0.001. **(C)** At effector: target ratio of 16:1, the cytotoxicity (%) of 4T1-Luc-HER2 group was compared with 4T1 group, ***P < 0.001. Three repeated tests were performed and the mean values were presented. CAR, chimeric antigen receptor; Luc, luciferase; CAR-T, T cells transduced with CAR; Blank T, T cells not transduced; LDH, lactate dehydrogenase.

In order to determine the efficacy of effector cells together with anti-PD1 antibody on target cells *in vitro*, anti-HER2 CAR-T cells were co-cultured with HER2-positive breast cancer cells (4T1-Luc-HER2) at effector: target ratios of 16:1, 8:1, 4:1 and 2:1 for 18 h in the absence or presence of anti-PD1 antibody. The cytotoxicity (%) of CAR-T group against the 4T1-Luc-HER2 cells was significantly higher than that of blank T group at every effector: target ratio (^*^P < 0.05 and ^***^P < 0.001) (Figure 6B). Addition of anti-PD1 antibody significantly enhanced the cytotoxicity (%) of CAR-T cells against 4T1-Luc-HER2 cells at the effector: target ratios of 16:1 and 8:1, as indicated by ^#^*P* < 0.05 (Figure 6B). No significant cytotoxicity differences were observed when HER2-negative 4T1 cells were used as the target cells (Figure 6A). At the effector: target ratio of 16:1, cytotoxicity (%) of CAR-T cells against 4T1-Luc-HER2 cells reached 39.8%, while cytotoxicity (%) against HER2-negtive 4T1 cells was much lower, at just 15.2% (Figure 6C) (*P* < 0.001). When anti-PD1 antibody was added, cytotoxicity (%) of CAR-T cells against 4T1-Luc-HER2 cells reached 49.5%, whereas cytotoxicity (%) against 4T1 cells remained low at just 16.4% (Figure 6C) (*P* < 0.001).

### Inhibition of Syngeneic 4T1 Tumor Growth by Anti-HER2 CAR-T Cells or Together With Anti-PD1 Antibody

**Figure 7:**
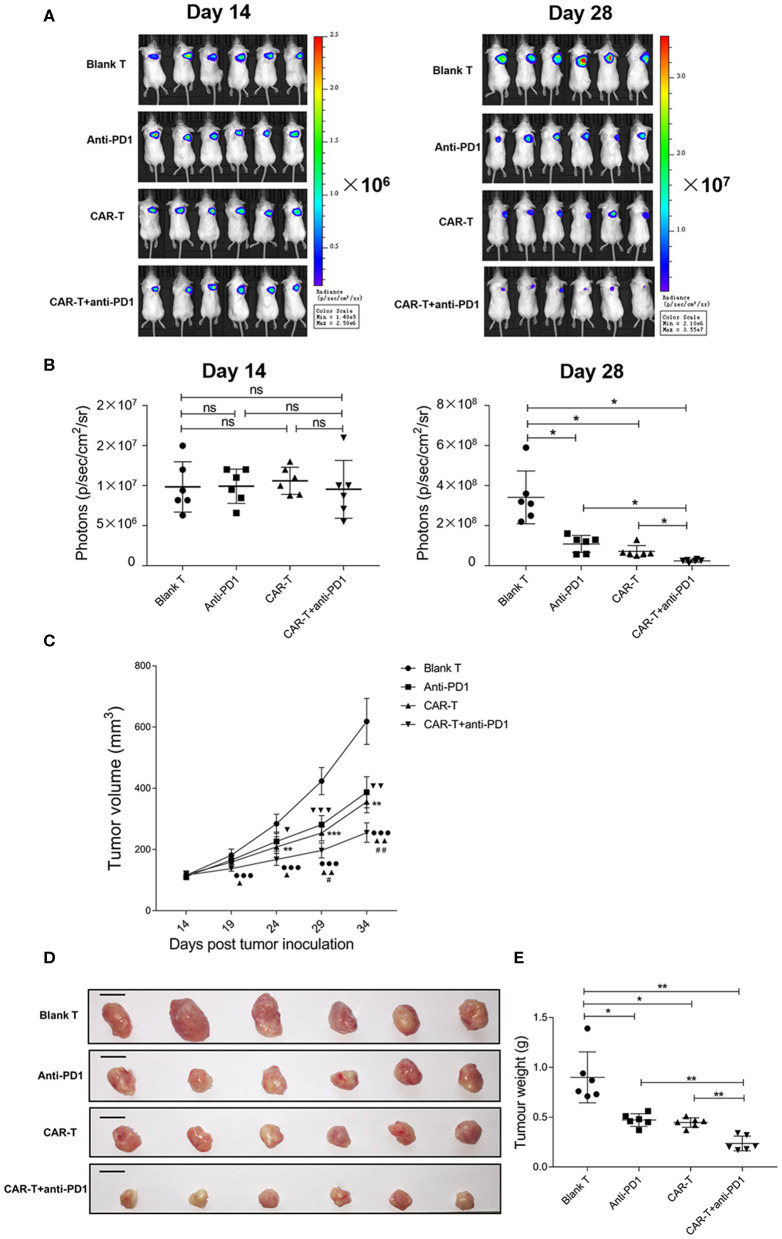
The inhibition of syngeneic tumor growth by anti-HER2 CAR-T cells or in combination with anti-PD1 antibody. **(A)** Tumor growth of mice was monitored by IVIS image, after the mice were injected with 4T1-Luc-HER2 cells for 2 weeks before anti-tumor treatment (on day 14), and after anti-tumor treatment for 2 weeks (on day 28). **(B)** Bioluminescence photons were monitored by IVIS image, after the mice were injected with 4T1-Luc-HER2 cells for 2 weeks before anti-tumor treatment (on day 14), and after anti-tumor treatment for 2 weeks (on day 28). CAR-T group compared with blank T group, or CAR-T plus anti-PD1 group compared with anti-PD1 group, or anti-PD1 group compared with blank T group, or CAR-T plus anti-PD1 group compared with CAR-T group, or CAR-T plus anti-PD1 group compared with blank T group, *ns:* not significant, *P < 0.05. **(C)** Tumor volumes (mm^3^) were calculated as described in “Materials and methods.” Three determinations were made and the mean values were shown. CAR-T group compared with blank T group, **P < 0.01, ***P < 0.001; CAR-T plus anti-PD1 group compared with anti-PD1 group, ▴*P* < 0.05, ▴▴*P* < 0.01; anti-PD1 group compared with blank T group, ▾*P* < 0.05, ▾▾*P* < 0.01, ▾▾▾*P* < 0.001; CAR-T plus anti-PD1 group compared with CAR-T group, ^#^*P* < 0.05, ^#^^#^*P* < 0.01; CAR-T plus anti-PD1 group compared with blank T group, •••*P* < 0.001. **(D)** The tumors were excised from tumor-bearing mice after anti-tumor treatment for 3 weeks (on day 35), the individual syngeneic tumor size was shown. Scale bar = 1 cm. **(E)** The excised tumors were weighed. CAR-T group compared with blank T group, or CAR-T plus anti-PD1 group compared with anti-PD1 group, or anti-PD1 group compared with blank T group, or CAR-T plus anti-PD1 group compared with CAR-T group, or CAR-T plus anti-PD1 group compared with blank T group, *P < 0.05, **P < 0.01. The experiments have been repeated for three times. CAR, chimeric antigen receptor; Luc, luciferase; CAR-T, T cells transduced with CAR; Blank T, T cells not transduced.

To examine whether anti-HER2 CAR-T cells alone or in combination with anti-PD1 antibody could inhibit the growth of HER2-positive tumors, we treated mice carrying the syngeneic 4T1-Luc-HER2 tumors by anti-HER2 CAR-T cells without or with anti-PD1 antibody. Tumor growth was monitored by IVIS system on day 14 and day 28. Bioluminescent imaging data revealed that there were not statistically significant among four groups on day 14 (just before anti-tumor treatment) (Figures 7A,B). On day 28 (after anti-tumor treatment for 14 days), CAR-T group significantly reduced the tumor growth compared with blank T group (*P* < 0.05) (Figures 7A,B). CAR-T plus anti-PD1 group had even higher inhibitory effect on tumor growth (*P* < 0.05) (Figures 7A,B).

The tumor volume of CAR-T group was reduced during the anti-tumor treatment compared with blank T group (^**^P < 0.01, ^***^P < 0.001), and the significant difference started on day 24 (after anti-tumor treatment for 10 days) (^**^P < 0.01) (Figure 7C). The tumor volume of CAR-T plus anti-PD1 group significantly reduced from day 29 (after anti-tumor treatment for 15 days) compared with CAR-T group (^#^*P* < 0.05) (Figure 7C).

The individual syngeneic tumor size of each group on day 35 was showed in Figure 7D, and the excised tumors were weighed. The tumor weight of CAR-T group was significantly less than those of blank T group (*P* < 0.05) (Figure 7E). CAR-T plus anti-PD1 group had the least tumor weight among four groups (Figure 7E). On day 35 (after anti-tumor treatment for 21 days), for the inhibition ratio of tumor weight, CAR-T group compared to blank T group was 50.0%, CAR-T plus anti-PD1 group compared to CAR-T group was 46.7%, CAR-T plus anti-PD1 group compared to blank T group was 73.3%. These results suggested that anti-HER2 CAR-T cells could shrink syngeneic tumors, and anti-PD1 antibody enhanced the anti-tumor activity of CAR-T cells *in vivo*.

### Histomorphometric Analysis of Syngeneic 4T1 Tumor Tissue After Anti-tumor Treatments

**Figure 8:**
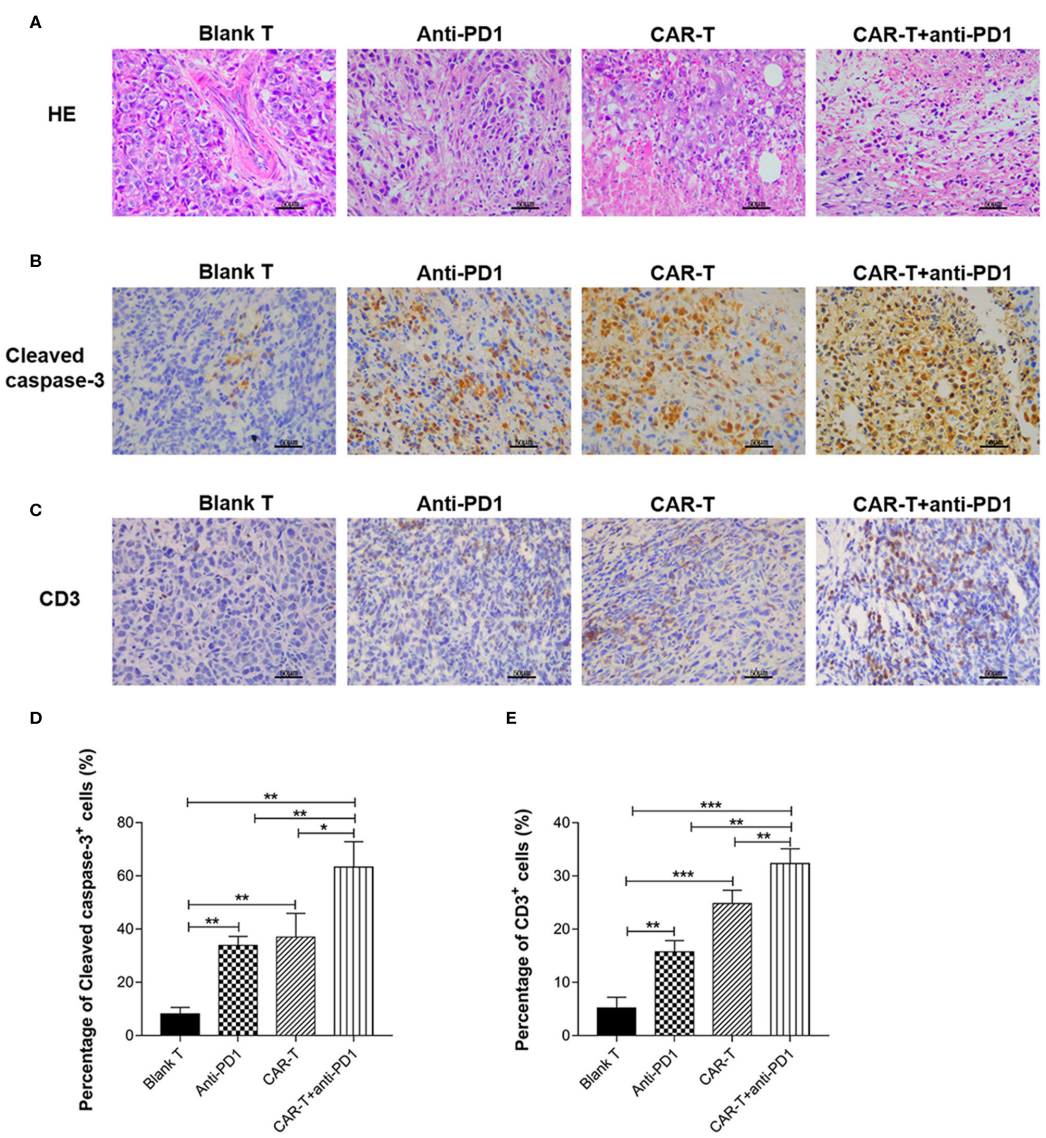
The syngeneic tumor tissue observed by microscope after HE staining or immunohistochemical staining and the quantification of cleaved caspase-3 and CD3 expression. **(A)** The tissue sections of syngeneic tumors were stained by HE and observed by microscope (400×). Scale bar is 50 μm. **(B)** The tissue sections (five tissue sections for each mouse) of syngeneic tumors were stained by immunohistochemistry for cleaved caspase-3 expression and observed by microscope (400×). The slides were incubated with rabbit anti-mouse cleaved caspase-3 antibody. Scale bar is 50 μm. **(C)** The tissue sections (five tissue sections for each mouse) of syngeneic tumors were stained by immunohistochemistry for CD3 expression and observed by microscope (400×). The slides were incubated with rabbit anti-mouse CD3ε antibody. Scale bar is 50 μm. **(D)** For quantification of immunohistochemical staining, cleaved caspase-3 positive cells were counted in five random 400× microscopic fields for each tissue section. **(E)** For quantification of immunohistochemical staining, CD3ε-positive cells were counted in five random 400× microscopic fields for each tissue section. CAR-T group compared with blank T group, or CAR-T plus anti-PD1 group compared with anti-PD1 group, or anti-PD1 group compared with blank T group, or CAR-T plus anti-PD1 group compared with CAR-T group, or CAR-T plus anti-PD1 group compared with blank T group, *P < 0.05, **P < 0.01, ***P < 0.001. The experiments have been repeated for three times. CAR, chimeric antigen receptor; Luc, luciferase; CAR-T, T cells transduced with CAR; Blank T, T cells not transduced.

HE stained slides of syngeneic tumor tissue were examined by an optical microscope (Figure 8A). The number of tumor cells was decreased significantly in the tissue sections from the CAR-T group and CAR-T plus anti-PD1 group compared with the blank-T group. Especially for CAR-T plus anti-PD1 group, nuclear/cytoplasm ratio was decreased; the cytoplasm mostly became transparent and filled with vacuoles, and more apoptotic cells had pyknotic nuclei. In contrast, in tumor sections from blank T group, there were more tumor cells that were densely organized. In addition, tumor cells were with significant larger and aberrant nuclei, and increased nuclear/cytoplasm ratio. Furthermore, the cytoplasm of tumor cells was rarely transparent and vacuous.

### Immunohistochemical Analysis of Cleaved Caspase-3 and CD3 Expression in Tumors From Syngeneic Mice After Anti-tumor Treatments

The positive staining of cleaved caspase-3 (as shown in brown) was localized mostly on cytoplasm and nucleus (Figure 8B) and the positive staining of CD3 (as shown in brown) was localized mostly on plasma membrane and cytoplasm (Figure 8C). Tumors from mice treated with blank T cells consisted primarily of living tumor cells, rarely of apoptotic tumor cells (cleaved caspase-3 staining), with occasional T cells (CD3 staining) (Figures 8B,C). There were more apoptotic tumor cells in the tumor tissues from CAR-T group (37.0%) than in those from blank T group (8.18%) (*P* < 0.01) (Figures 8B,D). Tumors from mice treated with CAR-T cells together with anti-PD1 antibody showed the highest percentage of apoptotic tumor cells (63.28%) among the four groups (Figures 8B,D). There were significantly more T cells in tumors from CAR-T group (24.8%) than in those from blank T group (5.18%) (*P* < 0.001 or *P* < 0.01) (Figures 8C,E). The highest T cell numbers were observed (32.34%) in tumors from mice treated with CAR-T cells together with anti-PD1 antibody (Figures 8C,E).

## Discussion

This study was about evaluating the anti-breast cancer efficacy of anti-HER2 CAR-T in combination with anti-PD1 antibody. Many previous studies have indicated that tumor specific CAR-T has therapeutic effect on target tumor. However, this effect was compromised by inhibitory signal delivered to CAR-T from target tumor cells through PD1-PDL1 signal pathway. Our hypothesis was blocking this signal pathway could enhance therapeutic effect of CAR-T. To prove this hypothesis, we first generated the mouse breast cancer 4T1-Luc-HER2 cells (Figure 1) from PDL1-positive 4T1 cells. 4T1-Luc-HER2 cells were ideal HER2^+^/PDL1^+^ target cells, since the percentage of HER2-positive (87.8%) and PDL1-positive (95%) was high (Figures 1A,C), and the expression level of HER2 protein was at least as same as that of HER2-positive human breast cancer BT474 and SKBR3 cells (Figure 1B). We then developed third-generation CAR containing CD28-CD137-CD3ζ (Figure 2A), which has two co-stimulatory molecules CD28 and 4-1BB/CD137. We prepared anti-HER2 CAR-T cells by transducing mouse spleen T cells with CAR-recombinant lentivirus (Figure 2). After successfully making our own HER2^+^/PDL1^+^ target tumor cells (4T1-Luc-HER2) and third generation anti-HER2 CAR-T cells, we tested the effects of anti-HER2 CAR-T cells on target tumor cells *in vitro* and *in vivo*, with or without combination of anti-PD1 antibody.

Our results showed that PD1 expression was increased in anti-HER2 CAR-T cells when co-cultured with HER2-positive breast cancer cells (Figure 3). HER2-positive breast cancer cells induced expansion of anti-HER2 CAR-T cells can be further enhanced with the addition of anti-PD1 antibody (Figure 4). Our results also showed that the secretion of IL-2 and IFN-γ from CAR-T cells was increased when co-cultured with 4T1-Luc-HER2 cells. Addition of the anti-PD1 antibody further enhanced the secretion of these two cytokines (Figure 5). Anti-HER2 CAR-T cells possessed potent cytotoxicity against 4T1-Luc-HER2 cells, and the addition of anti-PD1 antibody further enhanced the efficacy of CAR-T cells (Figure 6). At an effector: target of 16:1, after co-culture for 18 h, cytotoxicity (%) of CAR-T cells against 4T1-Luc-HER2 cells reached 39.8% (Figure 6C). With the addition of anti-PD1, cytotoxicity reached 49.5% (Figure 6C). All these results showed that addition of anti-PD1 antibody can enhance the efficacy of CAR-T cells against tumor cells *in vitro*. In the presence of the target tumor cells, CAR-T cells together with anti-PD1 antibody can secret more IL-2 and IFN-γ and eliminate tumor cells more efficiently in comparison with CAR-T cells alone. The results are consistent with our hypothesis that blocking PD1 signaling can rescue exhausted anti-HER2 CAR-T cells and can be a more effective treatment for cancer.

In most of the reported studies, NOD or severe combined immune deficiency (SCID) or NOD/SCID/γ-chain^−/−^ mouse xenograft tumor model was used to evaluate the efficacy of CAR-T cell treatment alone (37, 38) or in combination of PD1 blockage (27, 28, 30). However, the lack of a complete immune system in these mice precludes a detailed understanding of the complex tumor microenvironment that affects the overall efficacy of CAR-T cells or in combination of PD1 blockage. In order to simulate tumor microenvironment, in this study, we established a syngeneic tumor model using immune competent mice so that an intact immune system was considered. By using this immune competent tumor model, we found that anti-HER2 CAR-T cells could likely infiltrate into tumors (Figures 8C,E), inhibit growth (Figure 7), change histomorphology (Figure 8A), and increase apoptosis (Figures 8B,D) of HER2^+^ tumors. Adding anti-PD1 antibody could further enhance the anti-tumor activity of anti-HER2 CAR-T cells (Figures 7, 8). Twenty-one days after treatment (on day 35), tumor weight was reduced by 50.0% in CAR-T group compared with blank T group, and was decreased by 73.3% in CAR-T plus anti-PD1 group compared with blank T group. The tumors from mice treated with CAR-T cells in combination with anti-PD1 antibody showed the highest percentage of apoptotic tumor cells (63.28%) among the four groups (Figures 8B,D). There were significantly more T cells in tumors from CAR-T group than in those from blank T group (*P* < 0.001 or *P* < 0.01) (Figures 8C,E). The highest T cell numbers were observed in tumors from mice treated with CAR-T cells together with anti-PD1 antibody (Figures 8C,E). Theoretically mouse CD3-positive cells in the tumor stroma include all mouse endogenous and exogenous T cells. However, the more amount of CD3-positive cells in the tumor stroma of CAR-T group and CAR-T plus anti-PD1 group compared with blank T group were likely due to the presence of more CAR-T cells, based on our result *in vitro* that anti-HER2 CAR-T cells could expand better after co-cultured with HER2^+^ target cells, and the addition of anti-PD1 could further enhance their expansion.

Our study showed that the third generation anti-HER2 CAR-T cells can effectively eliminate HER2-positive breast tumor cells. The specificity of our anti-HER2 CAR-T cells relied on anti-HER2 scFv of CAR. The present study used the anti-HER2 scFv from specific anti-HER2 antibody 4D5 for CAR construction. The efficiency of our anti-HER2 CAR-T cells came from our selection of two co-stimulatory molecules CD28 and 4-1BB/CD137. The incorporation of additional co-stimulatory molecules into the CAR has greatly enhanced the efficacy of CAR-T cells. Various co-stimulatory molecules including CD28, 4-1BB/CD137, CD27, OX40 have been embedded in the CAR to explore their roles in antitumor immunity of CAR-T cells (39). In comparison with other co-stimulatory molecules, CD28 was more effective at increasing IL-2 production, enhancing clonal expansion and maintaining persistence of CAR-T cells. CD28 in combination with 4-1BB/CD137 is more effective than CD28 alone in eliciting cytotoxicity and IFN-γ production (40).

CAR-T cell therapy has become a promising method for the treatment of a variety of cancers (18–20, 41). However, the efficacy of CAR-T cell therapy for solid tumors was limited (18–20), one important reason was immunosuppressive microenvironment (20–22). PD1/PDL1 is one of the regulatory pathways that can serve as negative feedback loops after the initial immune response to switch off adaptive immunity (27). Currently, monoclonal antibody blocking PD1/PDL1 pathway has achieved encouraging results in patients in clinical trials (25, 26, 42). CAR-T cells in combination with immune inhibitors may improve the anti-tumor effect for solid tumors. The results in mouse models have indicated that anti-MSLN CAR-T (27) and anti-hPSMA CAR-T (28) cell therapy, in combination with anti-PD1 antibody, could improve the activity of CAR-T cells and enhance anti-tumor activity. The results from our *in vitro* and *in vivo* studies were consistent with these previous studies. PD1/PDL1 is only one of immunosuppressive pathways that affect the immune system against tumors. It will be interesting to see what will happen when we interfere with other immunosuppressive pathways.

In conclusion, our study demonstrated that third generation anti-HER2 CAR-T cells could specifically and efficiently eliminate HER2^+^ breast cancer cells *in vitro* and *in vivo*. When combined with anti-PD1 antibody, CAR-T cells had a stronger therapeutic effect on HER2^+^/PDL1^+^ breast cancer cells *in vitro* and in mouse model with an intact immune system.

## Data Availability Statement

All datasets generated for this study are included in the article/supplementary material.

## Ethics Statement

The animal study was reviewed and approved by the Institutional Animal Care and Use Committee of Wenzhou Medical University.

## Author Contributions

PL, LY, TL, SB, BS, and YH performed experiments. PL, LY, and KY analyzed the data. HL and HG conceived and supervised the study. HL, DS, HG, and PL drafted and wrote the manuscript. All authors contributed to the article and approved the submitted version.

## Conflict of Interest

The authors declare that the research was conducted in the absence of any commercial or financial relationships that could be construed as a potential conflict of interest.
